# Nicotine Facilitates Facial Stimulation-Evoked Mossy Fiber-Granule Cell Long-Term Potentiation *in vivo* in Mice

**DOI:** 10.3389/fncel.2022.905724

**Published:** 2022-07-04

**Authors:** Li-Xin Cao, Yan-Hua Bing, Yin-Hua Xu, Guang-Jian Zhang, Chun-Ping Chu, Lan Hong, De-Lai Qiu

**Affiliations:** ^1^Department of Physiology and Pathophysiology, College of Medicine, Yanbian University, Yanji, China; ^2^Department of Neurology, Affiliated Hospital of Yanbian University, Yanji, China; ^3^Department of Pain, Affiliated Hospital of Yanbian University, Yanji, China; ^4^Department of Physiology, College of Basic Medicine, Jilin Medical University, Jilin City, China

**Keywords:** cerebellar, facial stimulation, mossy fiber-granular cell, long-term potentiation (LTP), nicotine, neuropharmacology, electrophysiology

## Abstract

Nicotine is a psychoactive component of tobacco that plays critical roles in the regulation of neuronal circuit function and neuroplasticity and contributes to the improvement of working memory performance and motor learning function *via* nicotinic acetylcholine receptors (nAChRs). Under *in vivo* conditions, nicotine enhances facial stimulation-evoked mossy fiber-granule cell (MF-GrC) synaptic transmission, which suggests that nicotine regulates MF-GrC synaptic plasticity in the mouse cerebellar cortex. In this study, we investigated the effects of nicotine on facial stimulation-induced long-term potentiation (LTP) of MF-GrC synaptic transmission in urethane-anesthetized mice. Our results showed that facial stimulation at 20 Hz induced an MF-GrC LTP in the mouse cerebellar granular layer that was significantly enhanced by the application of nicotine (1 μM). Blockade of α4β2 nAChRs, but not α7 nAChRs, during delivery of 20 Hz facial stimulation prevented the nicotine-induced facilitation of MF-GrC LTP. Notably, the facial stimulation-induced MF-GrC LTP was abolished by an N-methyl-D-aspartate (NMDA) receptor antagonist, but it was restored by additional application of nicotine during delivery of 20 Hz facial stimulation. Furthermore, antagonism of α4β2 nAChRs, but not α7 nAChRs, during delivery of 20 Hz facial stimulation prevented nicotine-induced MF-GrC LTP. Moreover, inhibition of nitric oxide synthase (NOS) abolished the facial stimulation-induced MF-GrC LTP, as well as the effect of nicotine on it. Our results indicated that 20 Hz facial stimulation induced MF-GrC LTP *via* an NMDA receptor/nitric oxide (NO) cascade, but MF-GrC LTP was enhanced by nicotine through the α4β2 AChR/NO signaling pathway. These results suggest that nicotine-induced facilitation of MF-GrC LTP may play a critical role in the improvement of working memory performance and motor learning function.

## Introduction

Nicotine is the main psychoactive component of tobacco and plays an important role in the modulation of brain physiology *via* nicotinic acetylcholine receptors (nAChRs) (Dome et al., 2010). nAChRs are a family of ligand-gated ionic channels, which consist of α2–10 and β2–4 subunits (Gotti et al., 2006; Dani and Bertrand, 2007; Romanelli et al., 2007). A large number of studies have demonstrated that nicotine modulates neuroplasticity, including long-term depression (LTD) and long-term potentiation (LTP), which improves memory and motor learning function *via* nAChRs (Hahn et al., 2002; Kumari et al., 2003; Gonzalez et al., 2006; Heishman et al., 2010; Holschneider et al., 2011, 2013; Hijioka et al., 2012; Grundey et al., 2015; Grundey et al., 2018b). Administration of nicotine produces a focusing effect with an increase in focal LTP-like plasticity *via* a calcium-dependent mechanism (Kuo et al., 2007; Grundey et al., 2018a). Nicotine administration restores paired-associative stimulation-induced LTP-like excitatory neuroplasticity and improves the serial reaction time task performance in deprived smokers (Grundey et al., 2018b). Moreover, chronic administration of low-dose nicotine facilitates recovery and synaptic change after focal ischemia in rats (Gonzalez et al., 2006). Activation of α7 nAChRs or inhibition of cholinesterase improves motor behavior and sensorimotor performance in intracerebral hemorrhage (Hijioka et al., 2012) and ataxic model animals (Wecker et al., 2013). Previous studies have suggested that nicotine can improve motor behavior through modulation of neuronal circuitry function and long-term synaptic plasticity (Grundey et al., 2018b; Alhowail, 2021).

The cerebellar granule cells (GrCs) are assumed to act as a high-pass spatiotemporal filter; these cells receive information from mossy fibers (MFs) and convey them to Purkinje cells (PCs) *via* parallel fibers (D’Angelo and De Zeeuw, 2009; Solinas et al., 2010; Bing et al., 2015). The sensory information coming from MFs induces excitation of GrCs and long-term synaptic plasticity in the cerebellar granular layer (Roggeri et al., 2008; D’Angelo and De Zeeuw, 2009). We recently found that 20 Hz facial stimulation produced LTP of MF-GrC synaptic transmission through an NMDA receptor/nitric oxide (NO) signaling pathway (Lu et al., 2022). The sensory stimulation-induced long-term synaptic plasticity of MF-GrC synaptic transmission is assumed to play an important function during cerebellar adaptation to native MF excitatory inputs and motor learning behavior in living animals (Roggeri et al., 2008; D’Angelo and De Zeeuw, 2009; Lu et al., 2022). In addition, activation of nAChRs facilitates glutamate release at the MF-GrC synapses in cerebellar slices (De Filippi et al., 2001; Reno et al., 2004) and enhances facial stimulation-evoked MF-GrC synaptic transmission *via* activation of α7 and α4β2 nAChRs, which suggests that nicotine modulates sensory information-evoked MF-GrC long-term plasticity *via* nAChRs *in vivo* in mice (Xu et al., 2019). However, the effect of nicotine on MF-GrC long-term synaptic plasticity under *in vivo* conditions is unknown. In this study, we investigated the mechanism of nicotine-modulated facial stimulation-evoked MF-GrC LTP in urethane-anesthetized mice by an extracellular recording technique and pharmacological methods.

## Materials and Methods

### Anesthesia and Surgical Procedures

Anesthesia and surgical procedures have been described previously (Chu et al., 2011). A total of 88 (6- to 8-week-old) male HA/ICR mice were used in this study. The experimental procedures were approved by the Animal Care and Use Committee of Yanbian University and were in accordance with the animal welfare guidelines of the U.S. National Institutes of Health, and the Animal Research: Reporting *In Vivo* Experiments (ARRIVE^1^). The permit number is SYXK (Ji) 2011-006. All mice were housed under a 12-h light/12-h dark cycle with free access to food and water in a colony room under constant temperature (23 ± 1°C) and humidity (50 ± 5%). The mice were anesthetized with urethane (1.3 g/kg body weight, i.p.) and were tracheotomized for avoiding respiratory obstruction. The animals were fixed on a custom-made stereotaxic frame, and soft tissue was stripped to gain access to the dorsal portion of the occipital bone. A watertight recording chamber was created, and a 1- to 1.5-mm craniotomy was drilled to expose the cerebellar surface corresponding to Crus II. The brain surface was constantly superfused with oxygenated artificial cerebrospinal fluid (ACSF: 125 mM NaCl, 3 mM KCl, 1 mM MgSO_4_, 2 mM CaCl_2_, 1 mM NaH_2_PO_4_, 25 mM NaHCO_3_, and 10 mM d-glucose) with a peristaltic pump (Gilson Minipulse 3; Villiers, Le Bel, France) at 0.5 ml/min. Rectal temperature was monitored and maintained at 37.0 ± 0.2°C using body temperature equipment.

### Electrophysiological Recordings and Facial Stimulation

Local field potential recordings from the cerebellar granular layer were performed with an Axopatch 200B amplifier (Molecular Devices, Foster City, CA, United States) under current clamp conditions (*I* = 0) and acquired through a Digidata 1440 series analog-to-digital interface through a personal computer using Clampex 10.4 software. Recording electrodes were made with a puller (PB-10; Narishige, Tokyo, Japan) from thick-wall borosilicate glass, which were filled with ACSF and with resistances of 3–5 MΩ.

**FIGURE 1:**
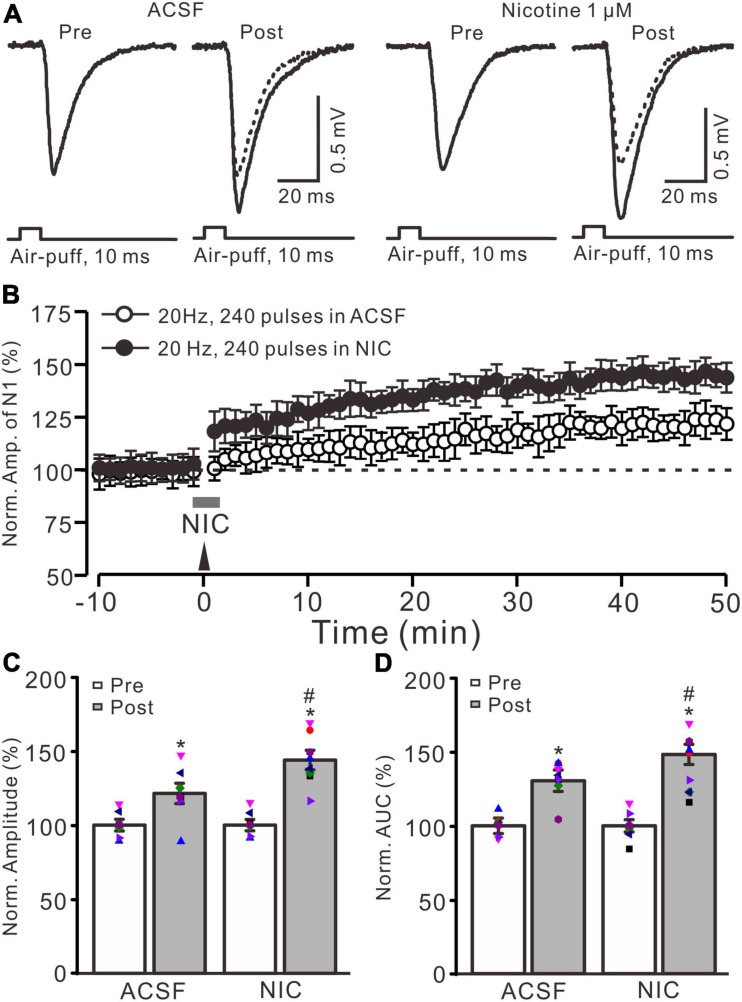
Nicotine enhanced the facial stimulation-induced LTP of MF-GrC synaptic transmission in the mouse cerebellum. **(A)** Representative extracellular recording traces illustrating facial stimulation (10 ms, 60 psi)-evoked MF-GC synaptic transmission before and after delivery of 20 Hz (240 pulses) stimulation in the absence (ACSF; open circle) and the presence (filled circle) of nicotine (NIC; 1 μM). **(B)** Summary of data showing the time course of normalized amplitude of the response before and after delivery of 20 Hz facial stimulation (arrow head) in the absence (ACSF; open circle) and the presence (filled circle) of nicotine (gray bar; NIC; 1 μM). **(C)** Bar graph showing the normalized amplitude before (pre) and after (post) delivery of 20 Hz stimulation in treatment of ACSF and nicotine (NIC; 1 μM). **(D)** Pooled data showing the normalized area under the curve (AUC) of the response before (pre) and after (post) delivery of 20 Hz stimulation in treatment of ACSF and nicotine (NIC). Note that facial stimulation at 20 Hz induced LTP of MF-GC synaptic transmission, which was enhanced by application of nicotine in the mouse cerebellar cortex. Data points are mean ± S.E.M. *n* = 8 recordings from eight mice in each group. *P < 0.05 vs. post; ^#^*P* < 0.05 vs. ACSF.

Facial stimulation was performed by air-puff (10 ms, 60 psi) of the ipsilateral whisker pad through a 12-gauge stainless steel tube connected with a pressurized injection system (Picospritzer^®^ III; Parker Hannifin Co., Pine Brook, NJ, United States). The air-puff stimulations were controlled by a personal computer and were synchronized with the electrophysiological recordings and delivered at 0.05 Hz *via* a Master 8 controller (A.M.P.I., Jerusalem, Israel) and Clampex 10.3 software. For obtaining MF-GC synaptic transmission, picrotoxin (100 μM) was added to ACSF during all recordings to prevent GABA_*A*_ receptor-mediated inhibitory component. In the presence of picrotoxin (100 μM), air-puff (10 ms, 60 psi) of the ipsilateral whisker pad evoked a negative component in granular layer of mouse cerebellar cortical folium Crus II (Figure 1A). According to our previous studies (Ma et al., 2019; Li et al., 2021; Lu et al., 2022), the facial stimulation-evoked response is identified as MF-GC synaptic transmission in the absence of GABAergic inhibitory inputs. For induction of long-term MF-GrC synaptic plasticity, 20 Hz air-puff stimulation (240 pulses) was delivered 10 min after facial stimulation-evoked response traces became stable (Lu et al., 2022).

### Chemicals

The reagents included picrotoxin, D-(-)-2-amino-5-phosphonopentanoic acid (D-APV), NG-Nitro-L-arginine (L-NNA), nitric oxide synthase (NOS) inhibitor, nicotine, methyllycaconitine citrate hydrate (MLA), and dihydro-β-erythroidine hydrobromide (DβEH) bought from Sigma-Aldrich (Shanghai, China). All these chemicals were dissolved in solution and kept in frozen aliquots. For experiments with NOS inhibitor, L-NNA, cerebellar surface was superfused with 200 μM L-NNA for 1 h before recordings were began. The drugs were dissolved in ACSF and applied directly onto the cerebellar surface by a peristaltic pump (0.5 ml/min).

### Statistical Analysis

The electrophysiological data were analyzed using Clampfit 10.6 software (Molecular Devices, Foster City, United States). The amplitude of the evoked field potential responses was maintained constant for an individual experiment before and after delivery of 20 Hz facial stimulation. Values are expressed as mean ± S.E.M. One-way ANOVA, followed by Tukey’s *post hoc* test (SPSS software; Chicago, IL, United States), was used to determine the level of statistical significance among groups of data. *P*-values below 0.05 were considered statistically significant.

## Results

### Cerebellar Surface Perfusion of Nicotine Enhanced the Facial Stimulation-Induced Mossy Fiber-Granule Cell Long-Term Potentiation *in vivo* in Mice

Blockade of GABA_*A*_ receptor-mediated inhibition by picrotoxin (100 μM) and facial stimulation with air-puff on the ipsilateral whisker pad induced a negative response in the cerebellar granular layer, which was identified as MF-GrC excitatory synaptic transmission (Ma et al., 2019; Li et al., 2021; Lu et al., 2022). Consistent with our previous study (Lu et al., 2022), facial stimulation at 20 Hz (240 pulse) induced LTP of MF-GrC synaptic transmission. As shown in Figure 1, delivery of 20 Hz facial stimulation induced a significant increase in the amplitude of response under control conditions for over 50 min (Figures 1A,B). During 40–50 min after 20 Hz facial stimulation, the normalized amplitude of response was 121.4 ± 6.9% of baseline (*F* = 32.8, *P* < 0.001; *n* = 8 experiments; Figure 1C), and the normalized area under the curve (AUC) of response was 130.5 ± 8.3% of baseline (*F* = 41.6, *P* < 0.001; *n* = 8 experiments; Figure 1). These results indicated that facial stimulation at 20 Hz induced MF-GrC LTP in the mouse cerebellar cortical Crus II.

Cerebellar surface perfusion of low-concentration nicotine (NIC; 1 μM) for 150 s did not change the amplitude of the facial stimulation-evoked response; the normalized amplitude of response was 100.2 ± 1.8% of baseline (ACSF; *F* = 0.06, *P* = 0.65). In the presence of nicotine, delivery of 20 Hz facial stimulation produced a stronger MF-GrC LTP than that under the control condition (Figures 1A,B). The normalized amplitude during 40–50 min after 1 Hz facial stimulation was 143.9 ± 6.6% of baseline (*F* = 56.1, *P* < 0.001; *n* = 8 experiments), which was significantly higher than that of the control group (121.4 ± 6.9% of baseline; *F* = 8.3, *P* = 0.016; *n* = 8 experiments; Figure 1C), and the normalized AUC of response was increased to 148.3 ± 7.5% of baseline (*F* = 48.5, *P* < 0.001; *n* = 8 experiments), which was significantly higher than that of the control group (ACSF; 130.5 ± 8.3% of baseline; *F* = 7.4, *P* = 0.021; *n* = 8 experiments; Figure 1D). These results indicate that application of nicotine facilitates the facial stimulation-induced MF-GrC LTP, which suggests that nicotine enhances MF-GrC LTP *via* activation of nAChRs *in vivo* in mice.

### Nicotine Enhanced the Facial Stimulation-Induced Mossy Fiber-Granule Cell Long-Term Potentiation *via* α4β2 Nicotinic Acetylcholine Receptors, but Not α7 Nicotinic Acetylcholine Receptors

**FIGURE 2:**
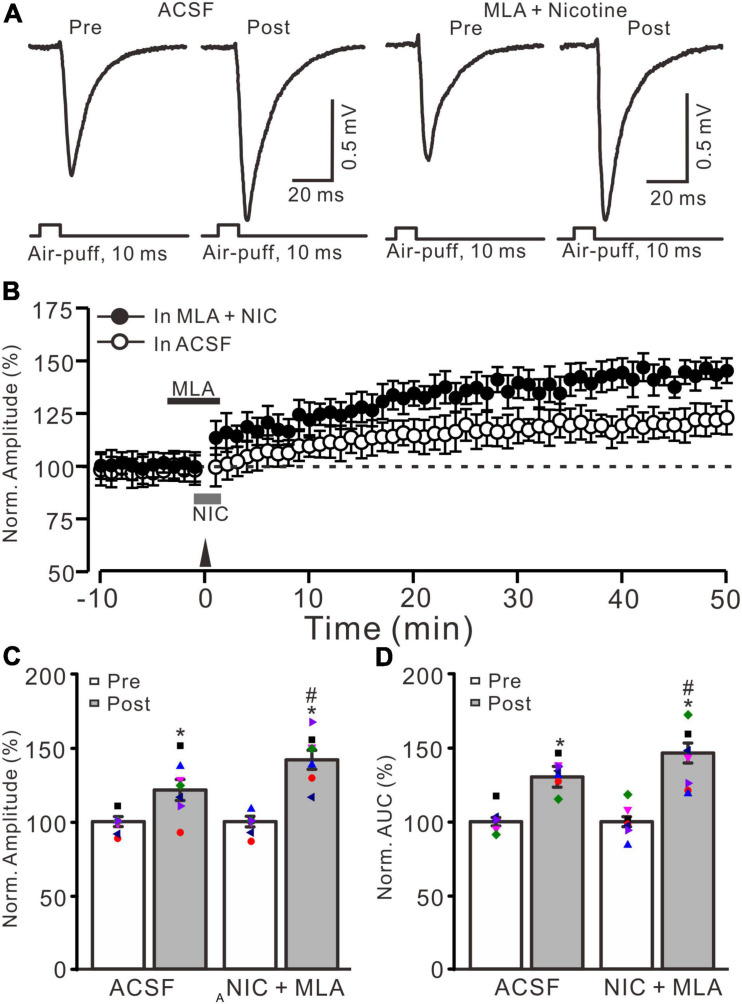
α7 nicotinic acetylcholine receptors antagonist failed to prevent the nicotine-induced enhancement of MF-GrC LTP. **(A)** Representative extracellular recording traces illustrating facial stimulation (10 ms, 60 psi)-evoked responses before and after delivery of 20 Hz (240 pulses) stimulation in the absence (ACSF) and the presence a mixture of α7 nAChR antagonist, MLA (1 μM), and nicotine (NIC; 1 μM). **(B)** Summary of data showing the time course of normalized amplitude of the response before and after delivery of 20 Hz facial stimulation (arrow head) in the absence (ACSF; open circle) and presence (filled circle) a mixture of MLA (1 μM) and nicotine (NIC; gray bar). **(C)** Bar graph showing normalized amplitude before (pre) and after (post) delivery of 20 Hz stimulation in treatment of ACSF and a mixture of MLA and nicotine (NIC). **(D)** Pooled data showing normalized AUC of the response before (pre) and after (post) delivery of 20 Hz stimulation in treatment of ACSF and a mixture of MLA and nicotine (NIC). Note that nicotine enhanced the facial stimulation induced MF-GrC LTP in the presence of α7-nAChR antagonist. Data points are mean ± S.E.M. *n* = 7 recordings from 7 mice in each group. *P < 0.05 vs. post; ^#^*P* < 0.05 vs. ACSF.

Because α7 nAChRs are expressed in presynaptic regions of the cerebellar granular layer and activation of these nAChRs enhances glutamate release at the MF-GrC synapses in rat cerebellar slices (De Filippi et al., 2001), we further employed a selective α7 nAChR antagonist, MLA (1 μM), to examine whether the nicotine-induced enhancement of MF-GrC LTP occurs through α7 nAChRs. To block α7 nAChRs, MLA was perfused to the cerebellar surface 150 s before the application of nicotine (Figure 2B). In the presence of a mixture of MLA (1 μM) and nicotine (1 μM), delivery of 20 Hz facial stimulation still induced a stronger MF-GrC LTP than in the control group (ACSF; Figure 2A), which expressed a strong time-dependent increase in amplitude of response (Figure 2B). During 40–50 min after 20 Hz facial stimulation, the normalized amplitude of response was 141.8 ± 6.4% of baseline (100.0 ± 4.7%; *F* = 62.3, *P* < 0.001; *n* = 7 experiments), which was significantly higher than that of the control group (121.4 ± 7.1% of baseline; *F* = 8.2, *P* = 0.013; *n* = 7 experiments; Figure 2C) but similar to that of nicotine alone (143.9 ± 6.6% of baseline; *F* = 0.21, *P* = 0.55; *n* = 7 experiments). The normalized AUC of response was 146.5 ± 6.8% of baseline, which was also significantly higher than that of the control group (130.4 ± 7.0% of baseline; *F* = 7.1, *P* = 0.017; *n* = 7 experiments; Figure 2C) but similar to that of nicotine alone (148.3 ± 7.5% of baseline; *F* = 0.18, *P* = 0.47; *n* = 7 experiments). These results indicate that blockade of α7 nAChRs failed to prevent the nicotine-induced enhancement of MF-GrC LTP, which suggests that nicotine enhancement of MF-GrC LTP is not dependent on α7 nAChRs.

**FIGURE 3:**
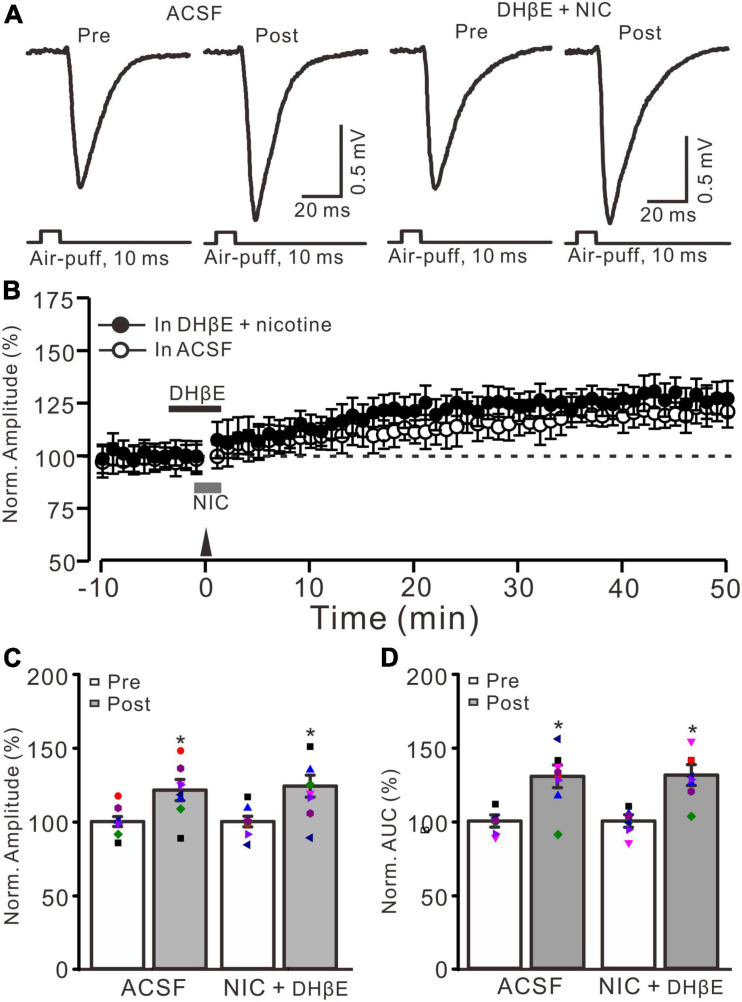
Nicotine-induced enhancement of MF-GrC LTP was abolished by α4β2 antagonist. **(A)** Representative extracellular recording traces illustrating facial stimulation (10 ms, 60 psi)-evoked MF-GrC synaptic response in a mouse cerebellar cortex before and after delivering 20 Hz (240 pulses) stimulation in the absence (ACSF) and the presence of a mixture of α4β2-nAChR blocker, DHβE (1 μM), and nicotine (NIC; 1 μM). **(B)** Summary of data showing the time course of normalized amplitude of the response before and after delivery of 20 Hz facial stimulation (arrow head) in the absence (ACSF, open circle) and the presence of a mixture of DhβE (black bar) and nicotine (NIC; gray bar) (filled circle). **(C)** Bar graph showing normalized amplitude of the response before (pre) and after (post) delivery of 20 Hz facial stimulation in the absence and the presence of a mixture of DHβE and nicotine. **(D)** Pooled data showing normalized AUC of the response before (pre) and after (post) delivery of 20 Hz facial stimulation in the absence and the presence of a mixture of DHβE and nicotine. Note that nicotine failed to enhance the facial stimulation-evoked MF-GrC LTP in the presence of α4β2 nAChR antagonist. Data points are mean ± S.E.M. *n* = 8 recordings from 8 mice in each group. *P < 0.05 vs. post.

In the cerebellar cortex, α4β2 nAChRs are present in the somas of GrCs and mediate somatic inward currents *in vitro* in rats (De Filippi et al., 2001). Therefore, we employed a selective α4β2 nAChR antagonist, DHβE (1 μM), to observe whether the nicotine-induced facilitation of MF-GrC LTP occurs *via* activation of α4β2 nAChRs. To block α4β2 nAChRs, DHβE (1 μM) was applied to the cerebellar surface 150 s before application of nicotine (Figure 3B). In the presence of a mixture of DHβE (1 μM) and nicotine (1 μM), enhancement of MG-GrC LTP was not induced by delivery of 20 Hz (240 pulses) facial stimulation (Figures 3A,B). In the presence of a mixture of DHβE and nicotine, the normalized amplitude of response was 124.1 ± 7.7% of baseline during 40–50 min after 20 Hz facial stimulation, which was similar to that of the control group (121.4 ± 7.2% of baseline; *F* = 0.15, *P* = 0.76; *n* = 8 experiments; Figure 3C), but significantly lower than that of nicotine alone (143.9 ± 6.6% of baseline; *F* = 8.37, *P* = 0.013; *n* = 8 experiments; not shown). In addition, the normalized AUC of response was 131.2 ± 7.1% of baseline during 40–50 min after 20 Hz facial stimulation, which was similar to that of control (ACSF: 130.4 ± 7.7% of baseline; *F* = 0.27, *P* = 0.61; *n* = 8 experiments; Figure 3D), but significantly lower than that of nicotine alone (148.3 ± 7.5% of baseline; *F* = 9.85, *P* = 0.009; *n* = 8 experiments). These results indicated that blockade of α4β2 nAChRs abolished the nicotine-induced facilitation of facial stimulation-induced MF-GrC LTP, which suggests that nicotine enhances MF-GrC LTP *via* activation of α4β2 nAChRs.

### N-Methyl-D-Aspartate Receptor Blockade Abolished Mossy Fiber-Granule Cell Long-Term Potentiation, but Nicotine Restored Mossy Fiber-Granule Cell Long-Term Potentiation *via* Activation of α4β2 Nicotinic Acetylcholine Receptors

**FIGURE 4:**
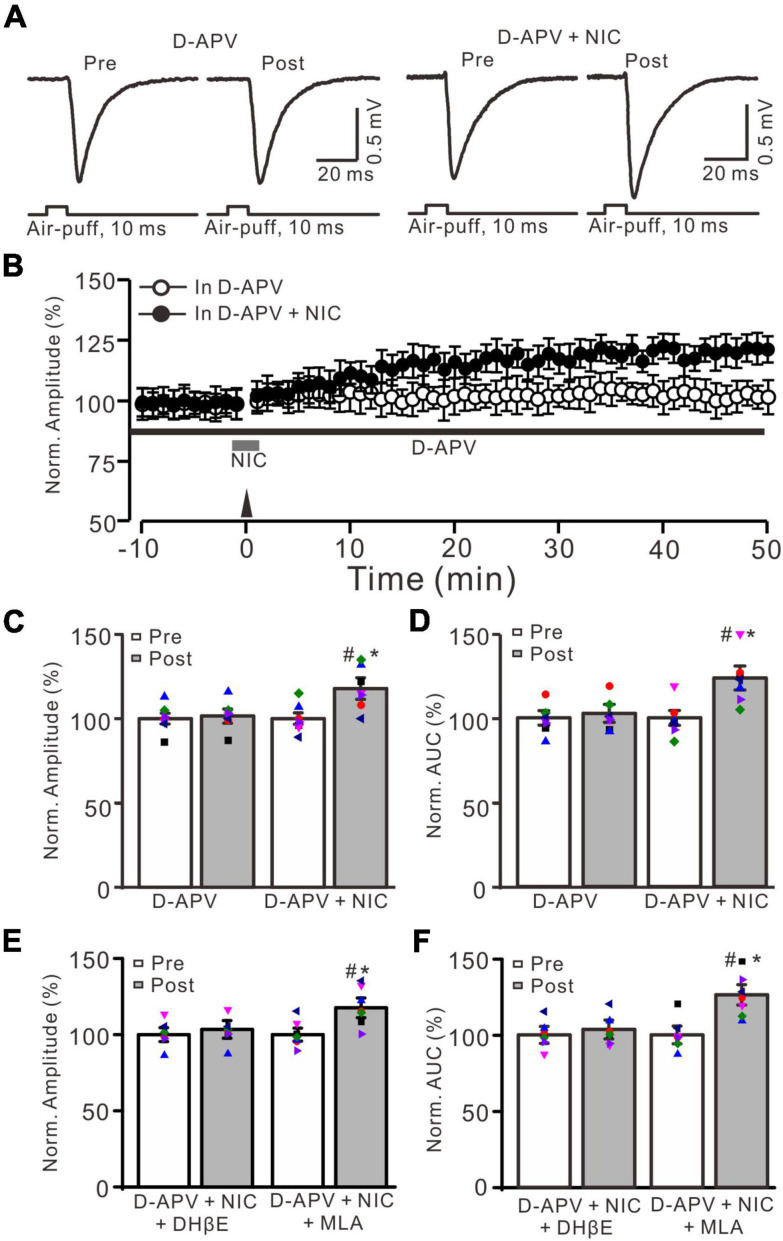
Facial stimulation-induced MF-GrC LTP was prevented by NMDA receptor antagonist, but it was restored by nicotine. **(A)** Representative extracellular recording traces illustrating facial stimulation (10 ms, 60 psi)-evoked MF-GrC synaptic response before (pre) and after (post) delivering 20 Hz (240 pulses) stimulation in the presence of D-APV (50 μM) and a mixture of D-APV + nicotine (D-APV + NIC; 1 μM). **(B)** Summary of data showing the time course of normalized amplitude before and after delivery of 20 Hz facial stimulation (arrow head) in the treatment with D-APV (black bar; open circle) and a mixture of D-APV + nicotine (NIC; gray bar; filled circle). **(C,D)** Bar graphs show normalized amplitude **(C)** and normalized AUC **(D)** of the response before (pre) and after (post) delivery of 20 Hz facial stimulation in treatment with D-APV and a mixture of D-APV + nicotine. Pooled data showing normalized amplitude **(E)** and normalized AUC **(F)** of the response before (pre) and after (post) delivery of 20 Hz facial stimulation in treatment with a mixture of D-APV (250 μM) + nicotine (1 μM) + DHβE (1 μM) (D-APV + NIC + DHβE), and a mixture of D-APV (250 μM) + nicotine (1 μM) + MLA (1 μM) (D-APV + NIC + MLA). Note that the facial stimulation-induced MF-GrC LTP was abolished by NMDA receptor blocker, D-APV, but it was restored by additional application of nicotine. The nicotine triggers the facial stimulation-induced MF-GrC LTP was abolished by DhβE, but not by MLA. Data points are mean ± S.E.M. *n* = 7 recordings from 7 mice in each group. *P < 0.05 vs. post; ^#^*P* < 0.05 vs. post of D-APV.

Our previous study showed that 20 Hz facial stimulation-induced MF-GrC LTP was dependent on the NMDA receptor. We further investigated whether nicotine could trigger MF-GrC LTP in the presence of the NMDA receptor antagonist. When the NMDA receptor was blocked with D-APV (250 μM), facial stimulation at 20 Hz could not induce MF-GrC LTP (Figures 4A,B). The normalized amplitude was 101.5 ± 4.2% of baseline during 45–50 min after 20 Hz facial stimulation (*F* = 0.71, *P* = 0.55; *n* = 7 experiments; Figure 4C), and the normalized AUC of response was 102.7 ± 5.3% of baseline during 40–50 min after 20 Hz facial stimulation (*F* = 0.75, *P* = 0.68; *n* = 7 experiments; Figure 4D). Notably, MF-GrC LTP was restored when co-application of D-APV and nicotine (1 μM) occurred during delivery of 20 Hz facial stimulation (Figure 4A). In the presence of a mixture of D-APV and nicotine, the normalized amplitude was 117.8 ± 6.4% of baseline during 45–50 min after 20 Hz facial stimulation (*F* = 45.4, *P* < 0.001; *n* = 7 experiments; Figure 4C), which was significantly higher than that of D-APV alone (*F* = 43.1, *P* < 0.001 versus D-APV; *n* = 7 experiments; Figure 4C). The normalized AUC was 123.7 ± 7.7% of baseline during 40–50 min after 20 Hz facial stimulation delivery in the presence of D-APV and nicotine (*F* = 52, *P* < 0.001 versus pre; *n* = 7 experiments; Figure 4D), which was significantly higher than that of D-APV alone (*F* = 48.3, *P* < 0.001 versus D-APV; *n* = 7 experiments; Figure 4D). However, upon blockade of α4β2 nAChRs, nicotine failed to trigger MF-GrC LTP. In the presence of a mixture of D-APV (250 μM), DhβE (1 μM), and nicotine (1 μM), the normalized amplitude was 103.4 ± 5.8% of baseline during 45–50 min after 20 Hz facial stimulation (*F* = 0.13, *P* = 0.51; *n* = 7 experiments; Figure 4E), and the normalized AUC was 103.6 ± 6.2% of baseline during 40–50 min after delivery of 20 Hz facial stimulation in the presence of D-APV and nicotine (*F* = 0.26, *P* = 0.62 versus pre; *n* = 7 experiments; Figure 4F). Moreover, blockade of α7 nAChRs did not prevent the nicotine-triggered MF-GrC LTP. In the presence of a mixture of D-APV (250 μM), MLA (1 μM), and nicotine (1 μM), the normalized amplitude was 117.6 ± 6.5% of baseline during 45–50 min after 20 Hz facial stimulation (*F* = 43.1, *P* < 0.001; *n* = 7 experiments; Figure 4E), and the normalized AUC was 126.3 ± 6.7% of baseline during 40–50 min after delivery of 20 Hz facial stimulation in the presence of D-APV and nicotine (*F* = 50.4, *P* < 0.001 versus pre; *n* = 7 experiments; Figure 4F). These results indicate that blockade of NMDA receptors abolished MF-GrC LTP, which was restored by nicotine through activation of α4β2 nAChRs.

**FIGURE 5:**
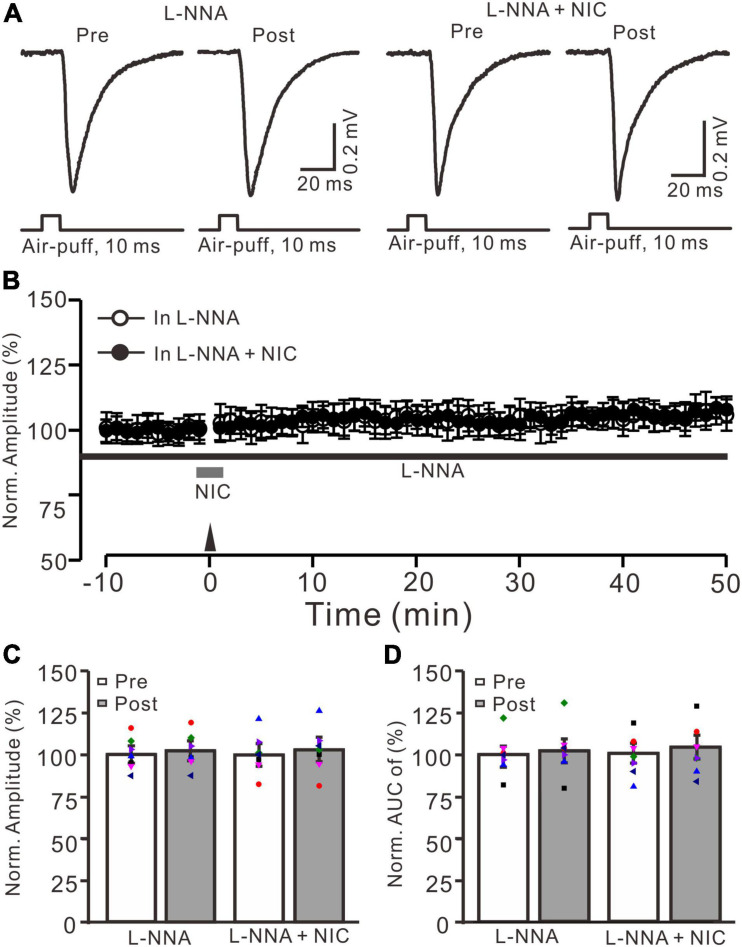
Nicotine-induced enhancement of facial stimulation-induced MF-GrC LTP was prevented by inhibition of NOS. **(A)** Representative extracellular recording traces illustrating facial stimulation (10 ms, 60 psi)-evoked MF-GrC synaptic response before (pre) and after (post) delivering 20 Hz (240 pulses) stimulation in the presence of NOS inhibitor, L-NNA (200 μM), and a mixture of L-NNA (200 μM) + nicotine (NIC; 1 μM). **(B)** Summary of data showing the time course of normalized amplitude before and after delivery of 20 Hz facial stimulation (arrow head) in the treatment with L-NNA (black bar; open circle) and a mixture of L-NNA + nicotine (NIC; gray bar; filled circle). **(C)** Bar graph showing normalized amplitude of the response before (pre) and after (post) delivery of 20 Hz facial stimulation in treatment with L-NNA and a mixture of L-NNA + nicotine (L-NNA + NIC). **(D)** Pooled data showing normalized AUC of the response before (pre) and after (post) delivery of 20 Hz facial stimulation in treatment with L-NNA and a mixture of L-NNA + nicotine (L-NNA + NIC). Note that the nicotine could not trigger MF-GrC LTP in the absence of NOS activity. Data points are mean ± S.E.M. *n* = 7 recordings from seven mice in each group.

Since facial stimulation at 20 Hz induced MF-GrC LTP in the mouse cerebellar granular layer through the NO cascade (Lu et al., 2022), we further examined whether nicotine enhances the facial stimulation-induced MF-GrC LTP *via* the NO signaling cascade. After perfusion of an NOS inhibitor, L-NNA (200 μM), on the cerebellar surface for 1 h, MF-GrC LTP could not be induced by delivery of 20 facial stimulation under the control condition (L-NNA) and in the presence of nicotine (NIC, 1 μM; L-NNA + NIC) (Figures 5A,B). In the presence of L-NNA, the normalized amplitude was 102.3 ± 4.4% of baseline during 45–50 min after 20 Hz facial stimulation (*F* = 0.093, *P* = 0.51; *n* = 7 experiments; Figure 5C), and the normalized AUC of response was 102.2 ± 6.4% of baseline during 40–50 min after 20 Hz facial stimulation (*F* = 0.11, *P* = 0.58; *n* = 7 experiments; Figure 5D). In the presence of a mixture of L-NNA and nicotine, the normalized amplitude was 102.7 ± 6.3% of baseline during 45–50 min after delivery of 20 Hz facial stimulation (*F* = 0.28, *P* = 0.45; *n* = 7 experiments; Figure 5C), and the normalized AUC of response was 103.4 ± 6.5% of baseline during 40–50 min after 20 Hz facial stimulation (*F* = 0.081, *P* = 0.58; *n* = 7 experiments; Figure 5D). These results indicate that inhibition of NOS abolished the facial stimulation-induced MF-GrC LTP and prevented the nicotine-restored LTP *in vivo* in mice. These results suggest that nicotine enhances MF-GrC LTP *via* the α4β2 nAChR/NO signaling pathway.

## Discussion

Cerebellar neuronal circuitry plasticity, including LTD and LTP is presumed to be a mechanism of motor learning (Ito, 1984). We recently found that facial stimulation evokes MF-GrC LTP *via* the NMDA receptor/NO signaling pathway, which suggests that the sensory stimulation-produced MF-GrC long-term plasticity contributes to cerebellar-related motor learning behavior (Lu et al., 2022). Nicotine plays important roles in modulation of cerebellar neuronal circuitry plasticity and improvement of motor functions *via* nAChRs. Our present results showed that 20 Hz facial stimulation induced an NMDA receptor-dependent MF-GrC LTP, which was significantly enhanced by nicotine *via* α4β2 nAChRs *in vivo* in mice. Blockade of NMDA receptor-dependent MF-GrC LTP revealed a nicotine-trigged MF-GrC LTP through α4β2-nAChR. However, neither the NMDA receptor-dependent nor nicotine-triggered MF-GrC LTP was abolished by inhibition of NOS. These results indicate that nicotine enhanced the MF-GrC LTP *via* the α4β2 nAChR/NO signaling pathway, which suggests that nicotine plays an important role in the modulation of motor learning behavior by regulating sensory-induced MF-GrC plasticity.

### Activation of Nicotinic Acetylcholine Receptors Modulate Neuronal Circuitry Activity in the Granular Layer

Nicotinic acetylcholine receptors are found abundantly in the central nervous system and contribute to modulate the release of other neurotransmitters and the synaptic transmission (Gotti et al., 2006; Dani and Bertrand, 2007; Romanelli et al., 2007). Previous studies have demonstrated that mRNAs of the α3, α4, α5, α7, α9, α10, β2, and β4 nAChR subunits are abundantly expressed in developing mouse cerebellar GrCs, and most GrCs express both α4 and β2 nAChRs (Didier et al., 1995; Tribollet et al., 2004; Turner and Kellar, 2005; Lykhmus et al., 2017). The α7 nAChRs are expressed in presynaptic sites of the cerebellar granular layer and contribute to facilitate the release of glutamate at the MF-GrC synapses *in vitro* in rats (De Filippi et al., 2001; Rossi et al., 2003; Reno et al., 2004). By contrast, the α4β2 nAChRs are present in the somas of cerebellar GrCs and contribute to modulate somatic membrane currents (De Filippi et al., 2001). Application of nicotine produces a concentration- and Ca^2+^-dependent GABA release in mouse cerebellar slices *via* activation of α7 and β2 nAChRs (Pereira da Silva et al., 2018). Cerebellar surface perfusion of nicotine induces an enhancement of facial stimulation-evoked field potential responses in the mouse cerebellar granular layer *via* activation of both α7 and α4β2 nAChR subunits, which suggests that nicotine modulates sensory stimulation-evoked MF-GrC synaptic transmission and plasticity *in vivo* in mice (Xu et al., 2019). Because the 50% effective concentration (EC_50_) of the nicotine-induced enhancement of the facial stimulation-evoked response in the granular layer is 32.6 μM (Xu et al., 2019), we employed low-concentration nicotine (1 μM) to avoid the direct action of nicotine on facial stimulation evoked MF-GrC synaptic transmission. Our data showed that application of 1 μM nicotine did not significantly change the amplitude of the response. However, delivery of 20 Hz facial stimulation in the presence of 1 μM nicotine induced an LTP of MF-GrC synaptic transmission in the mouse cerebellar cortex, which suggests that physiological activation of nAChRs is involved in facial stimulation-induced MF-GrC LTP *in vivo* in mice.

### Nicotine Enhances the Facial Stimulation-Induced Mossy Fiber-Granule Cell Long-Term Potentiation *via* α4β2 Nicotinic Acetylcholine Receptors

It has been well demonstrated that nicotine modulates neuroplasticity and improves working memory performance and motor function in both animals and human subjects (Hahn et al., 2002; Kumari et al., 2003; Heishman et al., 2010; Grundey et al., 2015; Toyoda, 2018, 2019). Application of nicotine produced a focusing effect with increased focal LTP-like plasticity induced by paired associative stimulation and abolished non-focal transcranial direct current stimulation-induced LTP-like plasticity *via* calcium-dependent mechanisms in healthy non-smokers (Kuo et al., 2007; Grundey et al., 2018b). Administration of nicotine restored the excitatory neuroplasticity and was linked to the improvement of implicit motor learning skills in deprived smokers (Grundey et al., 2018a). Furthermore, α4β2 nAChRs were present in the somas of cerebellar GrCs, which mediate somatic membrane currents *in vitro* in rats (De Filippi et al., 2001), and activation of α4β2 nAChRs facilitated long-term plasticity in the mouse insular cortex (Toyoda, 2018, 2019). Moreover, behavioral studies showed that application of nAChR agonists or cholinesterase inhibitor significantly improved the rotarod performance and increased functional activation of the cerebellar vermis in cortical impact injury or ataxic rats (Holschneider et al., 2011, 2013; Wecker et al., 2013). Our present results showed that in the presence of α4β2 nAChR antagonist, delivery of 20 Hz facial stimulation failed to enhance the MF-GrC LTP, which indicated that nicotine facilitated the facial stimulation-induced long-term synaptic plasticity in the cerebellar GrC layer *via* activation of α4β2 nAChRs. In addition, both α7 and α4β2 nAChRs contributed to modulate the facial stimulation-evoked MF-GrC synaptic transmission, but the present data showed that blockade of α7 nAChRs did not prevent the nicotine-induced enhancement of the MF-GrC LTP. The results indicate that nicotine facilitated the facial stimulation-induced MF-GrC LTP in the cerebellar granular layer, but this not occur through α7 nAChRs.

### Possible Mechanism of Nicotine-Induced Facilitation of Mossy Fiber-Granule Cell Long-Term Potentiation

**FIGURE 6:**
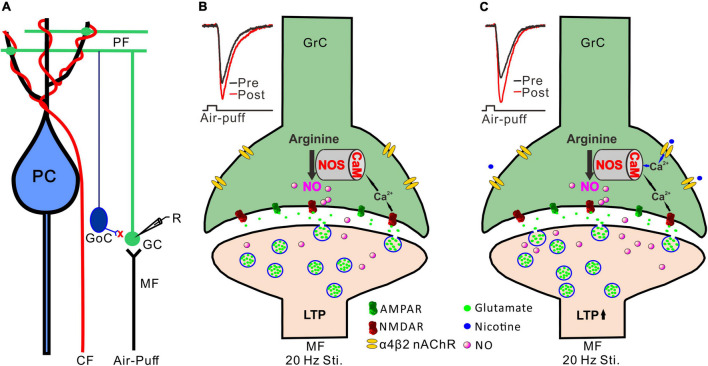
Possible mechanism of nicotine-induced facilitation of MF-GrC LTP. **(A)** Composition of cerebellar cortical microcirculation and the recording diagram of MF-GrC synaptic transmission evoked by facial stimulation. The inhibitory inputs of GoC were blocked by picrotoxin. **(B)** 20 Hz facial stimulation induced MF-GrC LTP *via* the NMDA receptor/NO cascade signaling pathway. Insert, representative traces showing the evoked MF-GrC synaptic transmission before (pre) and after (post) delivery of 20 Hz stimulation. **(C)** Activation of α4β2 nAChR enhanced Ca^2+^ influx and facilitated NO generation in GrC resulting in an enhancement of MF-GrC LTP expression. Insert, representative traces showing the evoked MF-GrC synaptic transmission before (pre) and after (post) delivery of 20 Hz stimulation. PC, Purkinje cell; CF, climbing fiber; MF-mossy fiber; GoC, Golgi cell; GrC, granule cell; R, recording electrode; NOS, nitric oxide synthase; NO, nitric oxide; LTP, long-term potentiation.

Activation of NMDA receptors is required for induction of long-term synaptic plasticity in the cerebellar granular layer (D’Angelo et al., 1999; Roggeri et al., 2008), including the 20 Hz facial stimulation-induced MF-GrC LTP (Lu et al., 2022). It has been proposed that 20 Hz facial stimulation induced Ca^2+^ influx from NMDA receptors and consequently activated calcium-sensitive protein and enzymes, such as calmodulin and NOS, resulting in an increase in NO levels (Kim and Huganir, 1999). Therefore, NO is generated by cerebellar GrCs during the 20 Hz facial stimulation *via* activation of NMDA receptors, resulting in MF-GrC LTP in the mouse cerebellar cortex (Figures 6A,B; Lu et al., 2022). Our present results showed that blockade of the NMDA receptor abolished the 20 Hz facial stimulation-induced MF-GrC LTP. However, upon blockade of NMDA receptor-dependent MF-GrC LTP, nicotine induced a kind of MF-GrC LTP through α4β2 nAChRs in response to 20 Hz facial stimulation. Importantly, we found that inhibition of NOS abolished the facial stimulation-induced LTP of MF-GrC synaptic transmission, regardless of the absence or presence of nicotine, which suggests that NOS activation was not only required for induction of the facial stimulation-induced MF-GrC LTP under control conditions but also necessary for the nicotine-induced enhancement of the LTP of MF-GrC synaptic transmission. Previous studies have demonstrated that chronic nicotine administration upregulates NOS expression in the hippocampus (Keser et al., 2011) and contributes to the improvement of motor coordination in female rats (Muñoz-Castañeda et al., 2014). Administration of nicotine significantly improves ethanol-induced impairment of memory, which is abolished by inhibition of NOS, suggesting that the effect of nicotine on ethanol-induced amnesia occurs *via* the NO signaling pathway in the CA1 regions of the dorsal hippocampus in adult mice (Raoufi et al., 2012). It is known that nicotine acts as an agonist of nAChRs with calcium channel properties and can increase intracellular calcium levels and transmitter release *via* both α7 and α4β2 nAChRs (Radcliffe and Dani, 1998; Fujii et al., 1999; Xu et al., 2019). Therefore, administration of nicotine during 20 Hz facial stimulation might induce an increase in calcium influx to achieve sufficient calcium levels and an enhancement of calmodulin activation, resulting in additional activation of NOS in the cerebellar cortical granular layer *via* α4β2 nAChRs (Figure 6C; Bredt and Snyder, 1990; Bredt et al., 1991; Garthwaite, 2019). Thus, nicotine facilitates NO release from cerebellar GrCs during 20 Hz facial stimulation *via* activation of α4β2 nAChRs, resulting in an enhancement of MF-GrC LTP in the mouse cerebellum (Garthwaite et al., 1988).

Long-term synaptic plasticity is a type of modification of synaptic strength that is considered to be a synaptic mechanism of motor learning (Ito, 1984). Notably, facial stimulation-evoked long-term synaptic plasticity in the cerebellar granular layer provides the spatiotemporal patterns of activity along the MF-GrC pathway, which is assumed to play important roles in motor coordination and motor learning behavior (Roggeri et al., 2008; D’Angelo and De Zeeuw, 2009; Solinas et al., 2010; Lu et al., 2022). Therefore, the present results may provide new insights for further understanding the mechanisms of nicotine-induced improvement of motor coordination and motor learning ability.

## Data Availability Statement

The original contributions presented in this study are included in the article/supplementary material, further inquiries can be directed to the corresponding authors.

## Ethics Statement

The animal study was reviewed and approved by the Animal Care and Use Committee of Yanbian University; the permit number is SYXK (Ji) 2011-006.

## Author Contributions

D-LQ, C-PC, and Y-HB conceived and designed the experiments. L-XC, Y-HX, and G-JZ performed the experiments. C-PC, Y-HB, and D-LQ analyzed the data. C-PC, LH and D-LQ wrote the manuscript. All authors contributed to the article and approved the submitted version.

## Conflict of Interest

The authors declare that the research was conducted in the absence of any commercial or financial relationships that could be construed as a potential conflict of interest.

## Publisher’s Note

All claims expressed in this article are solely those of the authors and do not necessarily represent those of their affiliated organizations, or those of the publisher, the editors and the reviewers. Any product that may be evaluated in this article, or claim that may be made by its manufacturer, is not guaranteed or endorsed by the publisher.

## Footnotes

1
https://arriveguidelines.org
